# Impacts of Different Grades of Tropical Cyclones on Infectious Diarrhea in Guangdong, 2005-2011

**DOI:** 10.1371/journal.pone.0131423

**Published:** 2015-06-24

**Authors:** Ruihua Kang, Huanmiao Xun, Ying Zhang, Wei Wang, Xin Wang, Baofa Jiang, Wei Ma

**Affiliations:** 1 Department of Epidemiology, School of Public Health, Shandong University, Jinan, Shandong, People’s Republic of China; 2 Shandong University Climate Change and Health Center, Jinan, Shandong, People’s Republic of China; 3 School of Public Health, University of Sydney, Sydney, Australia; Sun Yat-sen University, CHINA

## Abstract

**Objective:**

Guangdong province is one of the most vulnerable provinces to tropical cyclones in China. Most prior studies concentrated on the relationship between tropical cyclones and injuries and mortality. This study aimed to explore the impacts of different grades of tropical cyclones on infectious diarrhea incidence in Guangdong province, from 2005 to 2011.

**Methods:**

Mann-Whitney U test was firstly used to examine if infectious diarrhea were sensitive to tropical cyclone. Then unidirectional 1:1 case-crossover design was performed to quantitatively evaluate the relationship between daily number of infectious diarrhea and tropical cyclone from 2005 to 2011 in Guangdong, China. Principal component analysis (PCA) was applied to eliminate multicollinearity. Multivariate logistic regression model was used to estimate the hazard ratios (HRs) and the 95% confidence intervals (CI).

**Results:**

There were no significant relationships between tropical cyclone and bacillary dysentery, amebic dysentery, typhoid, and paratyphoid cases. Infectious diarrhea other than cholera, dysentery, typhoid and paratyphoid significantly increased after tropical cyclones. The strongest effect were shown on lag 1 day (HRs = 1.95, 95%CI = 1.22, 3.12) and no lagged effect was detected for tropical depression, tropical storm, severe tropical storm and typhoon, with the largest HRs (95%CI) of 2.16 (95%CI = 1.69, 2.76), 2.43 (95%CI = 1.65, 3.58) and 2.21 (95%CI = 1.65, 2.69), respectively. Among children below 5 years old, the impacts of all grades of tropical cyclones were strongest at lag 0 day. And HRs were 2.67 (95%CI = 1.10, 6.48), 2.49 (95%CI = 1.80, 3.44), 4.89 (95%CI = 2.37, 7.37) and 3.18 (95%CI = 2.10, 4.81), respectively.

**Conclusion:**

All grades of tropical cyclones could increase risk of other infectious diarrhea. Severe tropical storm has the strongest influence on other infectious diarrhea. The impacts of tropical cyclones on children under 5 years old were higher than total population.

## Introduction

Typhoon cyclone, a major disaster in China, has caused serious property and casualty losses and threatened sustainable development along the southeast coast of China [1]. According to the Chinese national standard of tropical cyclone category, tropical cyclones are categorized as tropical depression when the maximum average wind velocity near the bottom of tropical cyclone center reaches 10.8m/s-17.1m/s; tropical storm at 17.2 m/s-24.4 m/s; severe tropical storm at 24.5 m/s-32.6 m/s; typhoon at 32.7 m/s-41.4 m/s; severe typhoon at 41.5 m/s-50.9 m/s and super typhoon at 51.0 m/s [2]. Nearly all of the coastal provinces in China are affected by landing tropical cyclones every year [3]. Guangdong province is one of the provinces that most frequently hit by tropical cyclones. From 1949 to 2000, 477 tropical cyclones landed on China, among them, 203 landed on Guangdong with a mean annual frequency of 3.9 [4]. The landing tropical cyclones not only cause substantial direct economic losses but also threaten human health. For example, studies have reported the relationship between tropical cyclones and injuries and mortality [5–7].

In additional, tropical cyclone can also increase the risk of some communicable diseases, such as norovirus, cholera, Vibrio fluvialis, diarrhea and allergic diseases (asthma, allergic rhinitis, and atopic dermatitis) increased after tropical cyclones landed [8–12]. For example, following cyclone Aila, 3,529 (91.2%) of the 3,871 residents were affected by watery diarrhea within six weeks in Pakhirala village [8]. Another study in China indicated that typhoons and tropical storms increased the risk of bacillary dysentery and other infectious diarrhea in affected areas of Zhejiang province [13].

Among these diseases, infectious diarrhea remains an enormous global health problem. Diarrhea is usually a symptom of an infection in the intestinal tract, which can be caused by a variety of bacterial, viral and parasitic organisms [14]. Diarrheal disease is the second cause of death in children under five years old, and is responsible for killing approximately 760,000 children each year globally [14]. In 2011, 836,591 infectious diarrhea cases (other than cholera, dysentery, typhoid and paratyphoid) were reported, and the incidence rate was 62.39/100,000 in China. More than half patients were children aged under 5 year-old, accounting for 52.13% (436,098/836,591) and the incidence rate was 447.06/100,000 (436,098 cases)[15].

The increased infectious disease transmission and outbreaks are associated with the prolonged after-effects which include displaced people, environment changes, increasing vector breeding sites, high exposure, poor water and sanitation conditions, poor personal hygiene and limited access to healthcare services following natural disasters such as storms, typhoons, and hurricanes [16]. Tropical cyclones usually accompany with strong precipitation leading to flooding, increasing the probability of water contamination [17]. Because infectious diarrhea is mainly spread through fecal-oral routes, poor water and sanitation conditions play an important role in transmission. Hence, it is necessary to explore the association between tropical cyclones and infectious diarrhea.

There were few quantitative studies about the relationship between infectious diarrhea and tropical cyclones in China. In this study, we quantitatively examined the relationship between tropical cyclones and infectious diarrhea in landing cities of Guangdong province, from 2005 to 2011. Specifically, we also examined the relationship between tropical cyclones and occurrence of infectious diarrhea among “≤5 years” group, since this is a high risk population of infectious diarrhea.

## Materials and Methods

### Disease Data

Case report form of each notified patient with infectious diarrhea during tropical cyclone periods (from April to October) from 2005 to 2011 in Guangdong Province was obtained from the National Notifiable Disease Surveillance System (NDSS). According to the diagnostic criteria for infectious diarrhea in China, diarrhea is defined as the passage of three or more loose or liquid stools per day (or more frequent passage than is normal for the individual) [18]. NDSS defines infectious diarrhea as a group of human diseases that are mainly caused by microbes (including bacteria, parasites, and viruses) and have diarrhea as the typical symptom. Infectious diarrhea includes cholera, dysentery, typhoid, paratyphoid and other infectious diarrhea. Among these, cholera is a notifiable category A infectious disease in China and bacillary dysentery, amebic dysentery, typhoid and paratyphoid are notifiable category B infectious diseases. All bacillary dysentery, amebic dysentery, typhoid and paratyphoid cases were diagnosed by clinical syndromes and laboratory confirmation [18,19]. For infectious diarrhea other than cholera, dysentery, typhoid and paratyphoid, a term “other infectious diarrhea” is used and is categorized as a notifiable category C infectious disease. All reported other infectious diarrhea cases included clinical diagnosed cases and laboratory confirmed cases. The criteria of clinical diagnosed cases were diagnosed by physicians based on clinical syndromes and stool examination [20]. The criteria of laboratory confirmed cases covered stool examination and bacteriologic examination [20]. Common other infectious diarrhea includes salmonella enteritis, enteropathogenic Echerichia coli enteritis, enteropathogenic vibrio enteritis, Yersinia enterocolitica enteritis, rotaviruses enteritis, Norovirus Gastroenteritis, enteric adenovirus enteritis, Cryptosporidiosis and Giardiasis with incubation ranged from several hours to 2 weeks [20]. Physicians are obliged to report every case of above infectious diarrhea to the local health authority within 24 hours. Hence, it is believed that the degree of compliance in disease notification over the study period was consistent.

### Tropical Cyclones and Meteorological Data

Our study included all tropical cyclones which landed on Guangdong province from 2005 to 2011. Basic information of tropical cyclones was collected from the Yearbook of Tropical Cyclone. In this study, tropical cyclone was grouped into four levels as tropical depression, tropical storm, severe tropical storm and typhoon (including typhoon, severe typhoon and super typhoon, we use “typhoon” hereafter).

Daily meteorological data were obtained from the China Meteorological Data Sharing Service System (http://cdc.cma.gov.cn/). The meteorological variables included daily precipitation, daily average wind velocity, daily maximum wind velocity, daily extreme wind velocity, daily sunshine duration, daily average temperature, daily minimum temperature, daily maximum temperature, daily average relative humidity, daily minimum relative humidity, daily average air pressure, daily minimum air pressure, daily maximum air pressure and daily average vapor pressure. We used the meteorological data from meteorological station in the landing city or the nearest station if there is no meteorological surveillance station in the city of landfall.

### Study Areas and study population

**Fig 1:**
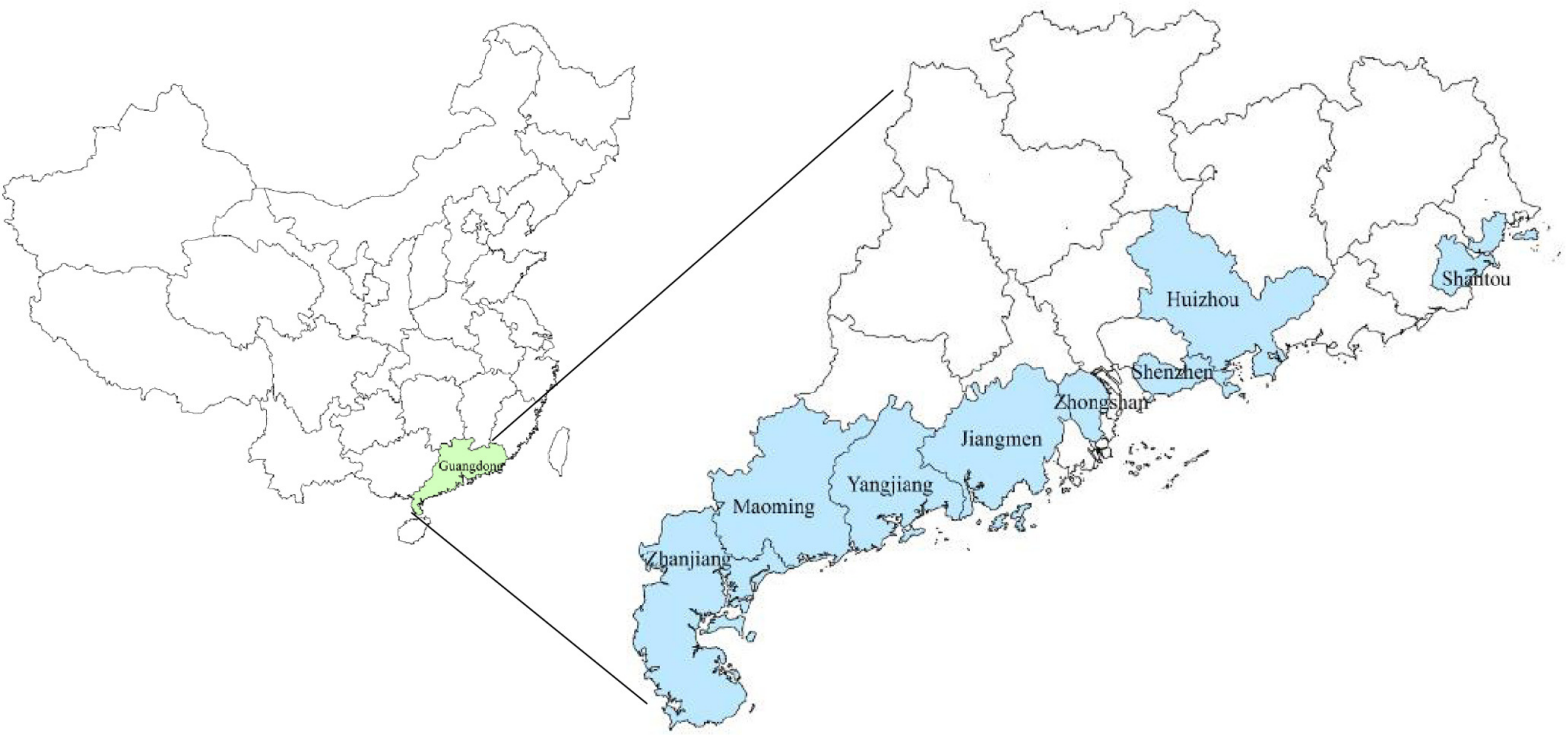
Study areas in Guangdong Province, China, from 2005 to 2011. All reported infectious diarrhea cases in landfall cities from 2005 to 2011 were included in this study. In addition, we analyzed the impacts of tropical cyclones upon occurrence of infectious diarrhea among high risk population of “≤ 5 years” group.

From 2005 to 2011, 19 tropical cyclones landed on Guangdong Province. As shown in Fig 1, they mainly landed on Shantou, Zhanjiang, Yangjiang, Shenzhen, Zhongshan, Maoming, Huizhou, and Jiangmen.

#### Study design and statistical analysis

Mann-Whitney U test was firstly used to examine if infectious diarrhea were sensitive to tropical cyclones. To evaluate the effects of tropical cyclones, we divided the period into 2 intervals for analysis. The early period (before the tropical cyclone) was five weeks before tropical cyclone landfall week. The latter period (after the tropical cyclone) included landfall week and subsequent four weeks.

**Fig 2:**
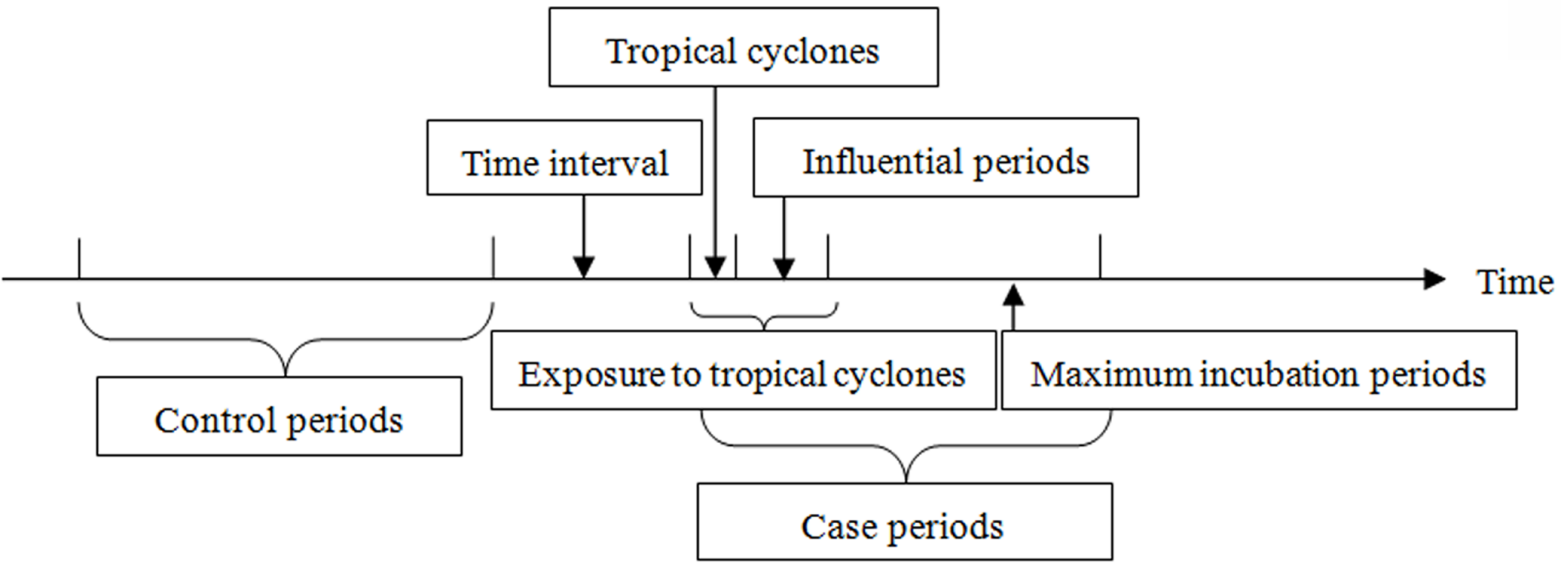
Case and control periods defined in the case-crossover design. In addition, principal component analysis (PCA) was used to eliminate the multicollinearity among meteorological factors. PCA was an effective method to reduce the dimensionality of a number of interrelated variables, while retaining the maximum variability in the data [22]. The standard of extracting principle components was that the eigenvector is larger than 1 and cumulative proportion reaches 70 percent to 85 percent. In this study, we confirmed 3 principle components. Then we tested the multicollinearity between tropical cyclone and three principle components and none was detected. Hence, tropical cyclone and three principle components were put into conditional logistic regression and hazard ratios (HRs) as well as 95% CI were calculated. Previous studies have reported a delayed onset of symptoms and subsequent hospitalization of diarrhea extreme precipitation [23]. Incubation periods of infectious diarrhea can range from one day (e.g., for Salmonella and Rotavirus) to up to one or two weeks (e.g., for Cryptosporidium, E.coli and Giardiasis) [20]. To account for the incubation periods, the 7-day lagged effects were considered in this study. All statistical analyses were performed using SAS 9.2 (SAS Institute Inc., USA).

Then unidirectional 1:1 case-crossover design was performed to evaluate the relationship between daily number of infectious diarrheas and tropical cyclones from 2005 to 2011.The case-crossover design was first described in 1991 by Maclure and it is an effective method to measure the influence of transient exposure to acute diseases [21]. This method combined with conditional logistic regression models is used to analyze the exposure effects of the case period compared with the control period. This design focused on the point of time when the event (tropical cyclone) occurred. The case periods included exposure to tropical cyclone and the maximum incubation period of infectious diarrhea. Control periods were two weeks before the first day of case periods and had the same week days with case periods. If the control period overlapped with case period, one or two prior weeks were chosen until all control days were not in the case period. Fig 2 showed that the case and control periods in this study.

### Ethical Statement

Disease surveillance data used in this study were obtained from the National Notifiable Disease Surveillance System (NDSS) with the approval by Chinese Center for Disease Control and Prevention. All identity information of patients were removed before data analysis. The study was approved by the Ethical Review Committee (ERC) of Public Health in Shandong University.

## Results

### Basic information of tropical cyclones and infectious diarrhea cases during study periods

From 2005 to 2011, 19 tropical cyclones landed on Guangdong province, consisting of 3 tropical depressions, 7 tropical storms, 3 severe tropical storms, 5 typhoons, and 1 severe typhoon. Details were in Table 1.

**Table 1:** Basic information of tropical cyclones landing on Guangdong province from 2005–2011.

Name	Landing city	Landing date	Exposure days	Grade
Sanvu	Shantou	13-Aug-05	4	Severe Tropical Strom
Chanchu	Shantou	18-May-06	2	Typhoon
Jelawat	Zhanjiang	29-Jun-06	1	Tropical Depression
Prapiroon	Yangjiang	3-Aug-06	2	Typhoon
Pabuk	Zhongshan	10-Aug-07	4	Tropical Strom
Neoguri	Yangjiang	19-Apr-08	2	Tropical Depression
Fengshen	Shenzhen	25-Jun-08	4	Tropical Strom
Kammuri	Yangjiang	6-Aug-08	4	Severe Tropical Strom
Nuri	Zhongshan	22-Aug-08	2	Severe Tropical Strom
Hagupit	Maoming	24-Sep-08	2	Super Typhoon
Higos	Zhanjiang	4-Oct-08	2	Tropical Depression
Nangka	Huizhou	26-Jun-09	3	Tropical Strom
Soudelor	Zhanjiang	12-Jul-09	1	Tropical Strom
Molave	Shenzhen	19-Jun-09	1	Typhoon
Goni	Jiangmen	5-Aug-09	2	Tropical Strom
Koppu	Jiangmen	15-Sep-09	2	Typhoon
Chanthu	Zhanjiang	22-Jul-10	3	Typhoon
Sarika	Shantou	11-Jun-11	2	Tropical Strom
Haima	Zhanjiang	23-Jun-11	3	Tropical Strom

Infectious diarrhea cases during tropical cyclone periods were shown in Table 2. From 2005 to 2011, there was no cholera case in Guangdong province. Other infectious diarrhea (infectious diarrhea other than cholera, dysentery, typhoid and paratyphoid) cases were the most frequent disease. Bacillary dysentery and amebic dysentery cases ranked the second, followed by typhoid and paratyphoid. The proportions were 86.76%, 10.95%, and 2.29% respectively.

**Table 2:** The number of infectious diarrhea cases during tropical cyclones periods (April to October) in Guangdong province from 2005 to 2011.

Diseases	2005	2006	2007	2008	2009	2010	2011	total
Cholera	0	0	0	0	0	0	0	0
Typhoid and paratyphoid	1447	1236	1207	1183	1240	1192	1074	8579
Bacillary dysentery and Amebic dysentery	7021	7566	7261	5949	5033	4564	3535	40929
Other infectious diarrhea	28858	43563	44890	46984	43238	55131	61754	324418
total	37326	52365	53358	54116	49511	60887	66363	373926

### Disease sensitive to tropical cyclones

The Mann-Whitney U test showed that there was no significant difference in weekly cases of dysentery, typhoid, and paratyphoid before and after 19 tropical cyclones in the landfall cities. Weekly cases of other infectious diarrhea after tropical cyclones were significantly higher than that before tropical cyclones in eight out of 19 tropical cyclones (Table 3).

**Table 3:** Relationship between tropical cyclones and infectious diarrhea in the landfall cities of Guangdong province, from 2005 to 2011 (*P* values of Mann-Whitney U test).

Tropical cyclone	Bacillary dysentery	Typhoid and paratyphoid	Other infectious diarrhea
Sanvu	0.594	0.591	0.401
Chanchu	0.084	0.455	0.016^*^
Jelawat	0.167	0.100	0.249
Prapiroon	0.915	0.221	0.075
Pabuk	0.013^*^	0.039	0.047^*^
Neoguri	0.554	1.000	0.434
Fengshen	0.600	1.000	0.047^*^
Kammuri	0.181	0.317	0.600
Nuri	0.456	0.217	0.916
Hagupi	0.399	0.513	0.047^*^
Higos	0.345	0.273	1.000
Nangka	0.828	0.650	0.011^*^
Soudelor	0.165	0.339	0.293
Molave	0.347	0.527	0.076
Goni	0.827	0.654	0.016^*^
Koppu	0.006^*^	0.074	0.016^*^
Chanthu	0.752	0.118	0.831
Sarika	0.234	0.164	0.009^*^
Haima	0.238	0.827	0.141

*P<0.05

### The influence of tropical cyclone on other infectious diarrhea

**Fig 3:**
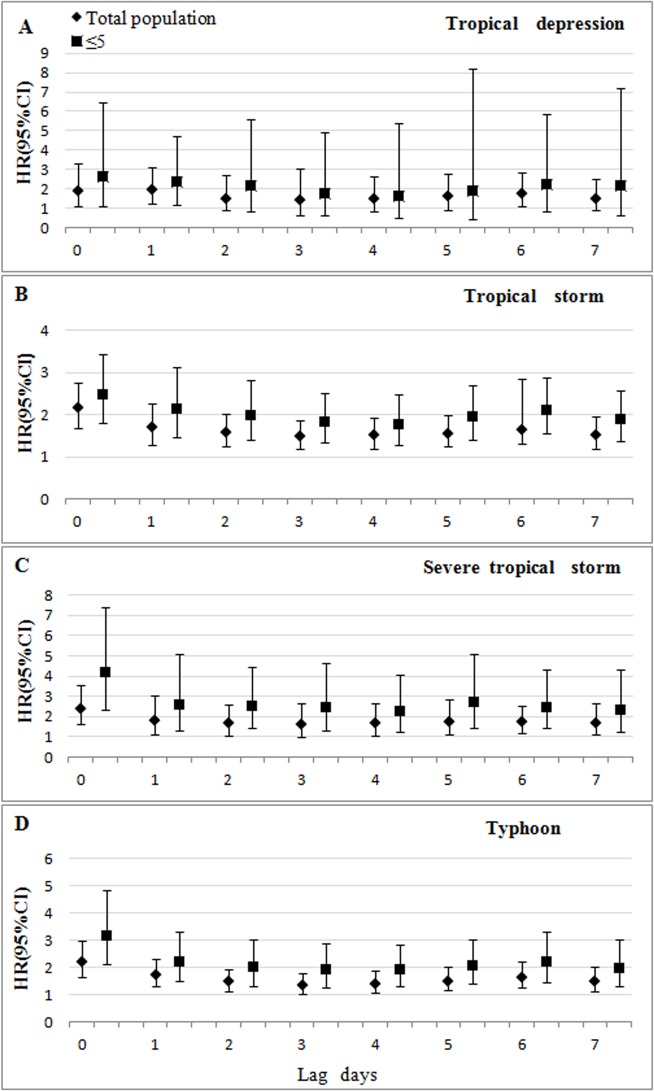
HRs of tropical cyclones on the risk of other infectious diarrhea on different lagged days. For total population, the impact of tropical depressions was largest at lag 1 day for other infectious diarrhea. The HR and 95% confidence interval was 1.95 (95%CI: 1.22, 3.12). The impacts of all tropical storms, severe tropical storms and typhoon were largest at lag 0 and HRs were 2.16 (95%CI = 1.69, 2.76), 2.43 (95%CI = 1.65, 3.58) and 2.21 (95%CI = 1.65, 2.69), respectively. For the “≤5 years” group, the influences of all tropical cyclones were largest at lag 0 for other infectious diarrhea. In another word, there were no delayed effects for tropical cyclones on other infectious diarrhea among “≤5 years” group. HRs were 2.67 (95%CI = 1.10, 6.48), 2.49 (95%CI = 1.80, 3.44), 4.89 (95%CI = 2.37, 7.37) and 3.18 (95%CI = 2.10, 4.81), respectively. Fig 3 also shows that the impacts of severe tropical storms were largest among all grades of tropical cyclones on other infectious diarrhea both for total population and “≤5 years” group.

Fig 3 shows that the impacts of tropical cyclones on other infectious diarrhea at lag 0–7 days among total population and high risk population. The significant effect of tropical depression, which was negative, occurred within lag 0–1, 6 and lag 0–1 among total population and “≤5 years” group respectively. For tropical storm, severe tropical storm and typhoon, we found significant increases in the incidence of infectious diarrhea among total population and “≤5 years” group at lag 0–7 days. The impact was stronger for “≤5 years” group than total population in all grades of tropical cyclones.

## Discussion

In this study, we found that tropical cyclones including tropical depression, tropical storm, severe tropical storm, typhoon increased the risk of other infectious diarrhea (infectious diarrhea other than cholera, dysentery, typhoid and paratyphoid) in landfall cities of Guangdong province of China. Our findings are consistent with previous studies. For example, severe cyclone storm Aila (the grade of Aila was equal to severe tropical storm), significantly increased 30%-60% incidence of diarrhea in 2009 in two subdivisions in India [8]. In South Korea, during the typhoon periods in 2003, cases of infectious diarrhea hospitalization significantly increased [24]. A study in China manifested that typhoons and tropical storms increased the risk of bacillary dysentery and other infectious diarrhea in Zhejiang province [13]. Another study in Myanmar, showed that atypical increases in diarrheal disease and especially dysentery cases occurred in 2008 following Cyclone Nagis compared to 2007 and 2009 [25]. Another study showed that cholera outbreak following cyclone Alia in Sundarban area of West Bengal, India, 2009[10]. Since there was no cholera case during our study periods, we could not confirm the relationship between tropical cyclones and cholera incidence in Guangdong. In addition, no significant relationship between tropical cyclones and dysentery was detected in our study. This may because there were few cases during the study periods. Hence, more researches are needed regarding the association between tropical cyclones and dysentery or cholera in China.

This study indicates that there are delayed effects of tropical cyclone on other infectious diarrhea. Tropical cyclones had different grades with different effects. In this study, we have detected lagged effects for tropical depressions but not for the other categories of typhoon on the other infectious diarrhea. The result was parallel with one study in South Korea, which also indicated typhoon could increase the hospitalization of infectious diarrhea without a lagged effect [24]. In USA, hurricane Katrina struck the Gulf Coast on August 29, 2005. Among evacuees of this typhoon, the number of acute gastroenteritis with diarrhea peaked on the seventh day after hurricane [26]. In Zhejiang province of China, the largest effect of typhoon and tropical storms was on lag 5 day and lag 6 day [13]. These studies had different delayed effects, which were biologically plausible because lag days were all within the incubation periods. Different lag effects in different districts and countries may due to different intensity and affected areas of typhoons, or different pathogen as well as different susceptibility of local populations, which need more researches to be done in the future.

WHO estimated the top six causes of death in children younger than 5 years globally and the diarrhea ranked the second [27]. Previous research in Guangdong found that the proportion of diarrheal in children under 5 years old was 63.5% and the morbidity of infectious diarrhea was 1,454.5/10,000 in 2012[28]. These researches indicated that children below 5 years old were the high risk population, which may be attributed to their weak immunity. Our study suggests that the influence of tropical cyclones on younger than 5 years were much greater to other infectious diarrhea than total population. Namely, children below 5 years were not only the high risk population of other infectious but also more vulnerable to other infectious diarrhea following tropical cyclone. Although the mechanism was not clear, the “≤5 years” group should be protected from touching insanitary water and food during tropical cyclone season, and be provided non-contaminated and nutritional food.

This study indicates that severe tropical storm has strongest influence on other infectious diarrhea, both for total population and “≤5 years” group. The reason might be that average exposure periods to tropical storms were longer than other grades. In this study, average exposure periods of severe tropical storms were 3.3 days, and average exposure periods of other grades of tropical cyclones were shorter than 3 days.

No lagged effects for tropical cyclones on infectious diarrhea were detected in this study. Therefore, a rapid infectious diseases risk assessment should be carried out within the first week of tropical cyclones in order to ascertain tropical cyclones impacts and health needs. Shelters, clean drinking water and food should be provided for victims timely.

Some strengths of the study included. First, the unidirectional 1:1 case-crossover design, which is similar to a 1:1 matched case-control study, could control some confounding factors, such as geographical characteristics, economic status, age, sex, behaviors, as cases were matched with themselves. Second, we considered the day of week effect in the control period selection for controlling potential time-trends. In addition, we analyzed impacts of different categories of tropical cyclones, which will provide scientific evidence for taking prevention and control measures after tropical cyclones landfall.

There are some limitations in our study. First, only meteorological factors were considered as confounders and analyzed through principle component analysis because of serious multicollinearity. There might be other factors that could influence transmission of infectious diarrhea. However, we believe the influence of other confounders is minimal because we used a case-crossover design. Second, the meteorological factors were not very accurate in some study areas without meteorological stations.

## Conclusion

Tropical cyclone could significantly increase the risk of infectious diarrhea other than cholera, dysentery, typhoid and paratyphoid in Guangdong. Tropical depression, tropical storm, severe tropical storm and typhoon have different impacts on other infectious diarrhea. Severe tropical storm has the strongest influence on other infectious diarrhea than other grades of tropical cyclones. Children less than 5 years old should be paid more attention during tropical cyclones seasons.
